# A Synergistic Strategy Combining Chemotherapy and Photodynamic Therapy to Eradicate Prostate Cancer

**DOI:** 10.3390/ijms25137086

**Published:** 2024-06-28

**Authors:** Aditi A. Shirke, Ethan Walker, Sriprada Chavali, Gopalakrishnan Ramamurthy, Lifang Zhang, Abhiram Panigrahi, James P. Basilion, Xinning Wang

**Affiliations:** 1 Department of Biomedical Engineering, Case Western Reserve University, 11100 Euclid Ave, Cleveland, OH 44106, USA; aas151@case.edu (A.A.S.); yvv@case.edu (E.W.); 2 Department of Biochemistry, Case Western Reserve University, 11100 Euclid Ave, Cleveland, OH 44106, USA; slc149@case.edu; 3 Department of Radiology, Case Western Reserve University, 11100 Euclid Ave, Cleveland, OH 44106, USA; gxr25@case.edu (G.R.); lxz749@case.edu (L.Z.); axp1084@case.edu (A.P.)

**Keywords:** photodynamic therapy, chemotherapy, combination treatment, PSMA, prostate cancer

## Abstract

Prostate cancer is the most prevalent cancer among men in the United States and is a leading cause of cancer-related death. Prostate specific membrane antigen (PSMA) has been established as a biomarker for prostate cancer diagnosis and treatment. This study aimed to develop a novel theranostic agent, PSMA-1-MMAE-Pc413, which integrates a PSMA-targeting ligand, the photosensitizer Pc413, and the microtubular inhibitor monomethyl auristatin E (MMAE) for synergistic therapeutic efficacy. In vitro uptake studies revealed that PSMA-1-MMAE-Pc413 demonstrated selective and specific uptake in PSMA-positive PC3pip cells but not in PSMA-negative PC3flu cells, with the uptake in PC3pip cells being approximately three times higher. In vitro cytotoxicity assays showed that, when exposed to light, PSMA-1-MMAE-Pc413 had a synergistic effect, leading to significantly greater cytotoxicity in PSMA-positive cells (IC_50_ = 2.2 nM) compared to PSMA-1-Pc413 with light irradiation (IC_50_ = 164.9 nM) or PSMA-1-MMAE-Pc413 without light irradiation (IC_50_ = 12.6 nM). In vivo imaging studies further demonstrated the selective uptake of PSMA-1-MMAE-Pc413 in PC3pip tumors. In in vivo studies, PSMA-1-MMAE-Pc413 dramatically improves the therapeutic outcome for prostate cancer by providing a synergistic effect that surpasses the efficacy of each treatment modality alone in PC3pip tumors. These findings suggest that PSMA-1-MMAE-Pc413 has strong potential for clinical application in improving prostate cancer treatment.

## 1. Introduction

Prostate cancer (PCa) is one of the most prevalent diseases in the world, with approximately over 1.4 million new cases and an estimated 37,500 deaths annually worldwide [1]. According to the American Cancer Society (ACS), PCa is the leading cancer in males in America with 288,000 new cases a year and 34,700 deaths annually [2]. Prostate cancer patients are usually diagnosed with prostate specific antigen (PSA) screening, biopsy, and magnetic resonance imaging (MRI). Notably, the incidence of PCa is particularly significant in older populations, with an average age of diagnosis at 66 years [3]. The anticipated rise in the prevalence of this disease is primarily attributed to the aging global population, coupled with various socioeconomic factors, making it a significant and pressing public health concern [4]. Many of the patients who die of this disease initially have cancer confined to the prostate gland and are identified in the high-risk localized prostate cancer category. These patients account for 15% of total diagnoses [5]. The standard approach often involves opting for radical prostatectomy as the primary mode of care with additional adjuvant and salvage therapies, such as radiation therapy, chemotherapy, or androgen deprivation therapy (ADT) [5,6]. However, these patients have a high risk of tumor recurrence following the treatment and will eventually develop lethal disease. This highlights the need for employing other more advanced methods of treatment to address the observed recurrence rates and enhance overall patient management. The main reason for the high rates of recurrence amongst high-risk localized prostate cancer is thought to be primarily due to the high heterogeneity of cancer cells [7,8]. The complexity of high-risk localized PCa, therefore, requires combined treatments. Furthermore, the use of novel molecular biomarkers in therapies has been observed to be more effective in treating aggressive forms of PCa [9,10]. It is imperative to develop molecularly targeted combination treatment options for prostate cancer. In doing so, a more personalized and nuanced approach can be achieved.

Prostate specific membrane antigen (PSMA) is a type II transmembrane protein found to be overexpressed in PCa as well as multiple solid human tumors [11,12,13]. It possesses several enzymatic functions that enhance processes such as angiogenesis, so higher PSMA expression often means poor prognosis in patients [14,15]. There have been many reports for PSMA-targeted imaging and therapeutic agents in PCa [16,17,18,19,20]. Among them, Locametz and Pylarify are approved by the Food and Drug Administration (FDA) for the positron emission tomography (PET) of PCa, and Pluvicto is approved for the radio-ligand treatment of metastatic castration-resistant prostate cancer (mCRPC).

Photodynamic therapy (PDT) stands as a minimally invasive approach crucial in battling cancers and other diseases such as retinopathy [21]. PDT involves the use of photosensitizers, which are initially inert compounds activated via light exposure in the presence of oxygen. Once activated, these agents unleash reactive oxygen species (ROS), notably singlet oxygen, effectively eradicating cells and disrupting tumor vasculature [22,23,24]. Additionally, PDT has been reported to stimulate an immune response, which can potentially prevent the recurrence of cancer [22,23,24]. Among the photosensitizers, compounds like porphyrins and phthalocyanines have been extensively explored as potent photosensitizers [25,26]. Some of these photosensitizers can emit fluorescence light and, therefore, can be used as a biomolecular imaging agent [27]. This imaging capability not only pinpoints affected areas but also facilitates fluorescence image-guided surgery and PDT, promising heightened precision and, consequently, enhanced therapeutic outcomes [28,29]. PDT’s killing effect can be localized when these compounds are targeted to a specific site, for example, through targeting with a tumor-associated antibody, or the application of light to a specific area. Such precision-targeted delivery systems hold the key to minimizing side effects and achieving superior therapeutic results.

Due to the distinctive mechanisms of PDT, it presents a more viable option for combination therapy with chemotherapy [30]. PDT can overcome chemotherapeutic drug resistance and increase drug uptake, while chemotherapy can kill cancer cells that have survived PDT. The combination of PDT with chemotherapy has shown high potency in vitro, in vivo, and in clinical trials [31,32]. There are two types of combination treatment. The first type is sequential, in which two drugs are administered separately [33,34]. The main difficulty with the approach is in finding the optimal administration time of each drug to achieve maximum synergism. Previously, we found that the co-injection of a PDT agent and a chemotherapeutic agent cannot improve antitumor activity [35]. To overcome this problem, a second strategy has been developed in which the two drugs are conjugated in one molecule and reach the tumor concomitantly [36]. In addition, this approach can provide drug accumulation information based on the fluorescence of the photosensitizer. This strategy can be further improved by including moiety targeting a cancer biomarker. We have previously reported one such multimodality molecule, PSMA-1-MMAE-IR700, which combines chemotherapeutic monomethyl auristatin E (MMAE) and photosensitizer IR700 in a single small molecule using a PSMA targeting ligand, PSMA-1 [35], and shows enhanced antitumor activity against PSMA-expressing tumors.

**Figure 1:**
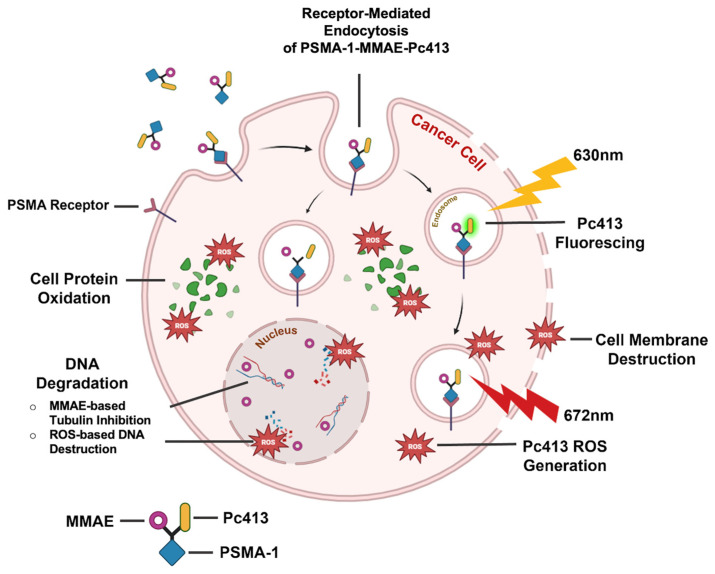
Mechanism of PSMA-1-MMAE-Pc413. PSMA-1-MMAE-Pc413 proves valuable for both visualizing and treating PCa, offering a multifaceted approach to these objectives. The Pc413 component serves as a theranostic agent, exhibiting fluorescence capabilities as well as functioning as a photosensitizer. Meanwhile, MMAE, a potent cytotoxic agent, will be released via cathepsin, further causing the destruction of PCa cells. [Illustration created with BioRender.com].

In our prior work, we introduced PSMA-targeted PDT agents, PSMA-1-Pc413 and PSMA-1-IR700 [37], demonstrating that they can provide effective PDT therapy alone and, using PSMA-1-Pc413, adjuvant PDT after fluorescence image-guided surgery (FIGS) resulted in successful surgical cures [29]. This surgical study highlights FIGS and PDT, specifically using PSMA-1-Pc413 as a promising strategy for enhancing surgical efficacy in the treatment of prostate cancer. Compared to IR700, Pc413 has higher fluorescence quantum yield (0.4) and singlet oxygen quantum yield (0.43) than those of IR700 (0.24 and 0.3, respectively) [28]. These properties prompted us to replace IR700 with Pc413 and develop PSMA-1-MMAE-Pc413. PSMA-1-MMAE-Pc413, as highlighted in Figure 1, targets cell surface PSMA, allowing for the uptake of this agent in prostate cancer cells through receptor-mediated endocytosis. At the peak time of tumor accumulation, fluorescence imaging enables the visualization of cancerous tissues. Subsequent PDT application initiates the generation of ROS, leading to the direct killing of tumor cells, the destruction of tumor-associated vasculature, and the initiation of an immune response. Moreover, as we have demonstrated before [35,38], MMAE will be released in active form by tumor-associated cathepsins and induce DNA damage, resulting in cell cycle arrest and attenuated tumor proliferation in cancer cells. With this method, we would be able to specifically target and visualize cancerous tissues with minimal cytotoxic effects on healthy tissues. Our results show that PSMA-1-MMAE-Pc413 was selectively delivered to PSMA-expressing cancer cells and the combination of Pc413 based-PDT and MMAE-based chemotherapy resulted in synergistic antitumor activity. The replacement of IR700 with Pc413 led to a more potent drug with reduced dose frequency, which will impact health outcomes and healthcare costs.

## 2. Results

### 2.1. Synthesis, Purification, and Validation of PSMA-1-MMAE-Pc413

**Figure 2:**
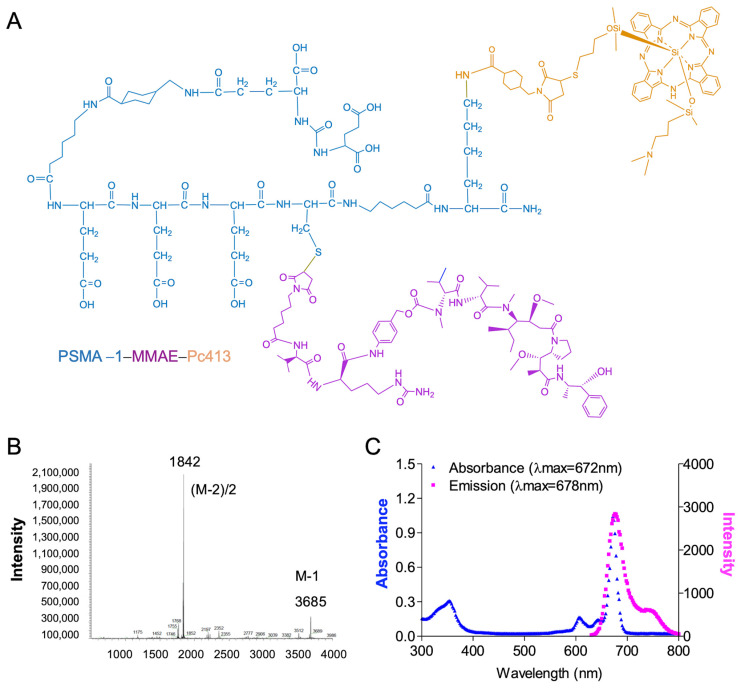
Characterization of PSMA-1-MMAE-Pc413. (**A**) Structure of PSMA-1-MMAE-Pc413. (**B**) MS spectrum of PSMA-1-MMAE-Pc413. (**C**) Absorbance (in blue) and emission (in pink) spectra of PSMA-1-MMAE-Pc413.

To synthesize, characterize, and validate PSMA-1-MMAE-Pc413, as shown in Figure 2A, we employed an established strategy used for synthesizing previous theranostic agents [35,37,38]. A cathepsin-cleavable linker, Vc linker, was used to conjugate MMAE to allow for the release of free MMAE, as we have demonstrated previously that the prodrug strategy is crucial for MMAE’s antitumor activity. The conjugation of Pc413 to the PSMA-1 ligand was achieved using a bifunctional SMCC linker as we did before for the synthesis of PSMA-1-Pc413 [37]. PSMA-1-MMAE-Pc413 was purified through high-pressure liquid chromatography (HPLC) and confirmed through mass spectra (MS); see Figure 2B. PSMA-1-MMAE-Pc413 had a maximum absorbance (λ_max_) at 672 nm—shown in Figure 2C—and emission at 678 nm, which matched with PSMA-1-Pc413, used in previous studies [37].

### 2.2. PSMA-1-MMAE-Pc413 Shows Specific Uptake and Enhanced Cytotoxicity to PSMA-Positive Cells Only

**Figure 3:**
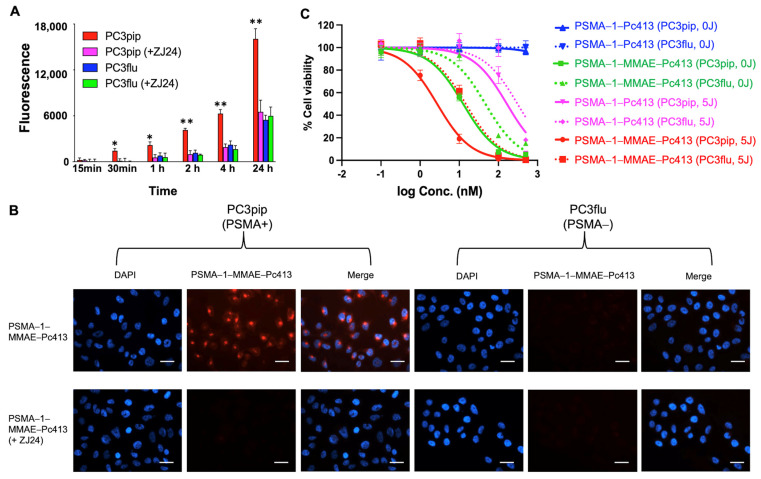
Uptake and cytotoxicity of PSMA-1-MMAE-Pc413. (**A**) Uptake of PSMA-1-MMAE-Pc413 in PC3pip and PC3flu cells at different incubation times. For blocking experiments, cells were co-incubated with a 10-fold excess amount of a known PSMA ligand ZJ24 [37,39]. Values are means ± SD of triplicates. *: *p* < 0.05, **: *p* < 0.01 (PC3pip cells vs. the other three groups). (**B**) Fluorescent images of PSMA-1-MMAE-Pc413 in PC3pip and PC3flu cells. A blocking experiment was performed in the presence of a 10-fold excess amount of ZJ24. DAPI is falsely colored blue and PSMA-1-MMAE-Pc413 is falsely colored red. Scale bar = 50 μm. Representative images are shown from three experiments. (**C**) Cytotoxicity assay of the treatments. Cells were incubated with drugs for 24 h. After that, cells were washed, treated with (5 J/cm^2^) or without 672 nm light (0 J/cm^2^), and incubated for another 24 h. Drug cytotoxicity was determined using a CellTiter 96 Aqueous One Solution Cell Proliferation Assay by measuring the absorbance at 490 nm. Values are means ± SD of six replicates.

To evaluate the selectivity and specificity of PSMA-1-MMAE-Pc413, uptake studies were conducted using both PSMA-positive PC3pip and PSMA-negative PC3flu cells over various time intervals. Additionally, PSMA ligand ZJ24 [37,39] was introduced to both cell populations to block PSMA receptors and validate the selective targeted uptake of the agent (Figure 3A). Notably, PSMA-positive PC3pip cells exhibited detectable fluorescence as early as 30 min, and the fluorescence signal consistently intensified over time, which was significantly higher as compared to both PSMA-negative PC3flu cells and PC3pip cells blocked with ZJ24. These data are presented visually in microscope images in Figure 3B. At 4 h, the top row of the left panel illustrates a distinct fluorescence signal exclusively observed in PC3pip cells, which diminishes upon co-incubation with ZJ24 (bottom row). In contrast, PSMA-negative PC3flu cells exhibit no uptake of PSMA-1-MMAE-Pc413 (right panel), and upon the addition of ZJ24, this lack of uptake is still evident with no signal being observed. These results demonstrated that PSMA-1-MMAE-Pc413 selectively targets PSMA.

We next performed a cytotoxicity assay including both PSMA-positive PC3pip and PSMA-negative PC3flu cells to examine whether the cytotoxicity of PSMA-1-MMAE-Pc413 is selective towards PSMA-positive cells (Figure 3C and Table S1). The cytotoxic effects of PSMA-1-MMAE-Pc413 and PSMA-1-Pc413 in PC3pip and PC3flu cells were first tested without light exposure (0 J/cm^2^) following a 24 h incubation. The cells were washed, and cytotoxicity was determined 24 h later using a CellTiter 96 Aqueous One Solution Cell Proliferation Assay. PSMA-1-Pc413 displayed no significant activity for either PC3pip or PC3flu cells, signifying that PSMA-1-Pc413 alone lacks toxicity in the absence of light activation. PSMA-1-MMAE-Pc413 without light irradiation showed considerable cytotoxicity of MMAE alone in PC3pip cells, IC_50_ = 12.6 ± 1.1 nM, compared to PC3flu cells, IC_50_ = 46.6 ± 3.2 nM (Figure 3C and Table S1). Upon addition of light, PSMA-1-Pc413 was activated and showed cytotoxicity with IC_50_ = 164.9 ± 33.1 nM for PC3pip cells and IC_50_ = 308.2 ± 46.5 nM for PC3flu cells (Figure 3C and Table S1). With light irradiation, PSMA-1-MMAE-Pc413 demonstrated a notably increased cytotoxicity in PC3pip cells, achieving an IC_50_ of 2.2 ± 0.4 nM (Figure 3C and Table S1). In contrast, its cytotoxicity to PC3flu cells was considerably lower, IC_50_ = 14.7 ± 0.5 nM. The cytotoxicity of PSMA-1-MMAE-Pc413 with light was considerably increased as compared to PSMA-1-Pc413 alone with light and PSMA-1-MMAE-Pc413 without light. The coefficient drug interaction (CDI) in PC3pip cells was 2.2/(12.6 × 164.9) = 0.001, less than 1, suggesting a synergistic effect resulting from the combination of PSMA-targeted PDT and MMAE [35,40]. In addition, the difference between the potency of the drug to PC3pip and PC3flu cells highlights that the selectivity of the cytotoxicity is linked to PSMA expression.

### 2.3. In Vivo Fluorescence Imaging Shows Selective Accumulation of PSMA-1-MMAE-Pc413 in PSMA-Positive Tumors

**Figure 4:**
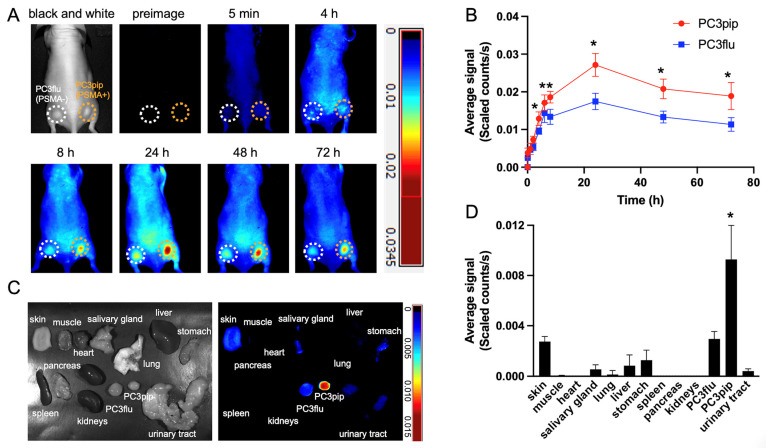
In vivo fluorescence images of PSMA-1-MMAE-Pc413 in mice. Mice received 100 nmol/kg of PSMA-1-MMAE-Pc413 intravenously. (**A**) Maestro images of mice bearing PC3pip (orange circle) and PC3flu (white circle) tumors. Images shown are representative images of 5 mice. (**B**) Quantification of average fluorescent signals on tumors. *: *p* < 0.05 (PC3pip vs. PC3flu). Values are means ± SD of 5 animals. (**C**) Ex vivo images of PSMA-1-MMAE-Pc413 at 72 h post-injection. Images shown are representative images of 5 mice. (**D**) Quantification of average fluorescent signals on organs. *: *p* < 0.01 (PC3pip vs. all other organs). Values are means ± SD of 5 animals.

To evaluate the selectivity of PSMA-1-MMAE-Pc413 in vivo, fluorescence imaging was conducted in mice harboring both PC3pip and PC3flu tumors. PSMA-1-MMAE-Pc413 (100 nmol/kg) was delivered via tail vein injection and mice were imaged at various timepoints, shown in Figure 4A, and fluorescence signals from both PC3pip and PC3flu tumors were quantified and graphed; see Figure 4B. A significantly higher selective uptake in PSMA-positive PC3pip tumors was observed as compared to PSMA-negative PC3flu tumors with signals peaking at 24 h post-injection. Upon the extraction of organs at 72 h post-injection, a significantly higher uptake in PC3pip tumors was observed in comparison to PC3flu tissues as well as other healthy tissues in the same mouse; see Figure 4C,D. This dataset validates the selective uptake of PSMA-1-MMAE-Pc413 in PSMA-positive tissues. A brief pharmacokinetic study was performed by measuring fluorescence signals in mouse plasma (Figure S1). The blood half-life (t_1/2_) of PSMA-1-MMAE-Pc413 was 7.55 ± 0.91 h.

### 2.4. PSMA-1-MMAE-Pc413 with PDT Demonstrated Synergistic Antitumor Activity In Vivo

**Figure 5:**
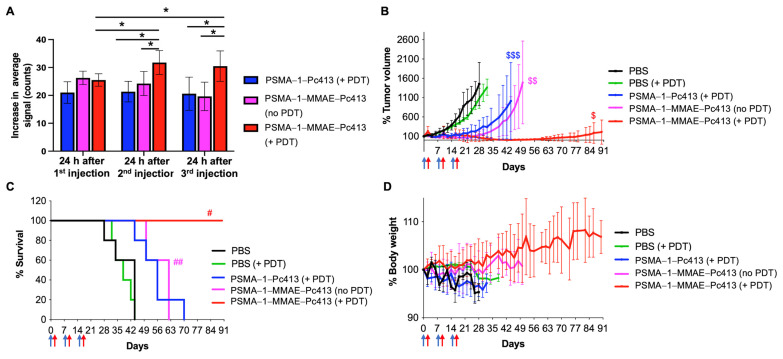
In vivo treatment of PC3pip tumors with PSMA-1-MMAE-Pc413. Mice received 100 nmol/kg of drug every 7 days, and PDT was performed at 24 h after each injection. (**A**) Increase in average fluorescent signals on PC3pip tumors after each injection. Values are means ± SD of 5 animals. *: *p* < 0.05. (**B**) Tumor growth curves of mice received different treatments. Values are means ± SD of 5 animals. $: *p* < 0.05, PSMA-1-MMAE-Pc413 + PDT vs. all other groups; $$: PSMA-1-MMAE-Pc413 with no PDT to PBS and PBS + PDT groups; $$$: PSMA-1-Pc413 + PDT to PBS and PBS + PDT groups. Blue arrows indicate drug administration time and red arrows indicate PDT application time. (**C**) Kaplan–Meier survival curves of animals. Blue arrows indicate drug administration time and red arrows indicate PDT application time. #: *p* < 0.05, PSMA-1-MMAE-Pc413 + PDT vs. all other groups; ##: PSMA-1-MMAE-Pc413 with no PDT to PBS and PBS + PDT groups. (**D**) Body weight changes in mice received different treatments. Blue arrows indicate drug administration time and red arrows indicate PDT application time. Values are means ± SD of 5 animals.

The efficacy of PSMA-1-MMAE-Pc413 in eradicating prostate tumors was assessed in male athymic nude mice inoculated with PC3pip tumors intravenously injected (i.v.) with different agents. Several experimental conditions were assessed: (i) PBS with untreated control (PBS); (ii) PBS with 150 J/cm^2^ of 672 nm light to test the effect of light on tumor growth (PBS + PDT); (iii) PSMA-1-Pc413 100 nmol/kg with 150 J/cm^2^ of 672 nm light to test the PDT effect of PSMA-1-Pc413 on tumor growth (PSMA-1-Pc413 + PDT); (iv) PSMA-1-MMAE-Pc413 100 nmol/kg to test the chemotherapeutic effect of PSMA-1-MMAE-Pc413 on tumor growth (PSMA-1-MMAE-Pc413 with no PDT); and (v) PSMA-1-MMAE-Pc413 100 nmol/kg with 150 J/cm^2^ of 672 nm light to test the combination treatment of PDT and chemotherapy on tumor growth (PSMA-1-MMAE-Pc413 + PDT). Mice received a dose of drugs every 7 days for a total of three doses, and PDT was performed 24 h after each injection. The 24 h timepoint was chosen based on the peak tumor accumulation time from in vivo imaging studies. Mice that received PBS had minimal background fluorescence. At 24 h post-injection of PSMA-1-Pc413 or PSMA-1-MMAE-Pc413, intense fluorescence was observed in PC3pip tumors and the fluorescence signal in PC3pip tumors was diminished after light irradiation, indicating the activation of Pc413 via light (Figure S2). The quantification of fluorescence on PC3pip tumors showed that at 24 h after the first injection, the fluorescence signal on PC3pip tumors was about the same for the mice receiving PSMA-1-Pc413 or PSMA-1-MMAE-Pc413 (Figure S2 and Figure 5A). Twenty-four hours after the second and third injections, the fluorescence signal increase in the PSMA-1-Pc413 + PDT group and the PSMA-1-MMAE-Pc413 with no PDT group was similar to the signal increase measured after the first injection; see Figure 5A. However, in the PSMA-1-MMAE-Pc413 + PDT group, there was an increase in fluorescence signal measured for PC3pip after the second (31.8 ± 4.3 counts) and the third injections (30.5 ± 5.4 counts). This increase was significantly higher than those measured after the first injection of PSMA-1-MMAE-Pc413 in both the + PDT group (25.5 ± 2.2 counts) and no-PDT group (26.3 ± 3.9 counts), and those measured after the second and third injections in the PSMA-1-Pc413 + PDT group (21.4 ± 3.7 counts and 20.6 ± 5.9 counts, respectively) and the PSMA-1-MMAE-Pc413 with no PDT group (24.3 ± 4.3 counts and 17.6 ± 4.2 counts, respectively). These data indicate that PDT can improve the subsequent uptake of PSMA-1-MMAE-Pc413. This finding is similar to the results we have found in which gold nanoparticles were used to deliver photosensitizer Pc158 to tumor cells and following PDT showed an increase in the subsequent uptake of gold nanoparticles [41].

For mice that received PBS and light irradiation with 150 J/cm^2^ of 672 nm light, no inhibitory activity was observed, indicating that light-only treatment does not affect tumor growth or animal survival (*p* > 0.9999 between PBS and PBS + PDT) (Figure 5B,C and Tables S2–S4). Compared to the PBS group, PSMA-1-Pc413 with PDT decreased tumor growth rate significantly (*p* = 0.0457). Although PSMA-1-Pc413 with PDT inhibited tumor growth and prolonged animal survival time, the survival benefit from the treatment was not significant as compared to the PBS group (*p* = 0.1905) (Figure 5C, Tables S3 and S4). PSMA-1-MMAE-Pc413 without PDT significantly inhibited tumor growth rate (*p* = 0.0058 vs. PBS) and extended survival times (*p* = 0.0079 vs. PBS), showing that MMAE itself is a potent cytotoxic agent. No significant difference in tumor growth rates (*p* = 0.0999) and animal survival times (*p* = 0.2143) were observed between the PSMA-1-Pc413 + PDT group and the PSMA-1-MMAE-Pc413 with no PDT group. None of the mice in PSMA-1-Pc413 + PDT and PSMA-1-MMAE-Pc413 with no PDT treatment groups was tumor-free and all mice in these groups died before the 90-day experimental time, indicating PDT or chemotherapy treatment alone is not effective in eradicating the tumors. In contrast, in the group that received PSMA-1-MMAE-Pc413 with PDT, all five mice were tumor-free from days 36 to 58. Although two mice had tumors grow back afterwards, they all survived the 90-day experimental time, resulting in a mean survival time of 90 days (Figure 5B,C and Tables S2–S4). Three mice remained tumor-free by the end of the 90-day observation period, achieving a 60% cure. Hence, the application of light significantly enhanced the therapeutic efficacy of PSMA-1-MMAE-Pc413 compared to PSMA-1-MMAE-Pc413 without light irradiation or PSMA-1-Pc413 with light irradiation, demonstrating the improved efficacy of combination treatment. On day 28 after the treatment, tumors in the PBS group grew 1370 ± 468%, and those in PSMA-1-Pc413 + PDT, PSMA-1-MMAE-Pc413 without PDT, and PSMA-1-MMAE-Pc413 + PDT grew 342 ± 275%, 188 ± 131%, and 37 ± 53% (only two tumors regrew), respectively. The CDI value was (37 ÷ 1370)/[(342 ÷ 1370) × (188 ÷ 1370)] = 0.79, which is less than 1, indicating that combination treatment results in synergism in vivo. During the treatment, no loss of body weight was observed in these treatment groups, suggesting the treatments were well tolerated. (Figure 5D).

### 2.5. PSMA-1-MMAE-Pc413 with PDT Caused More Tissue Damage

**Figure 6:**
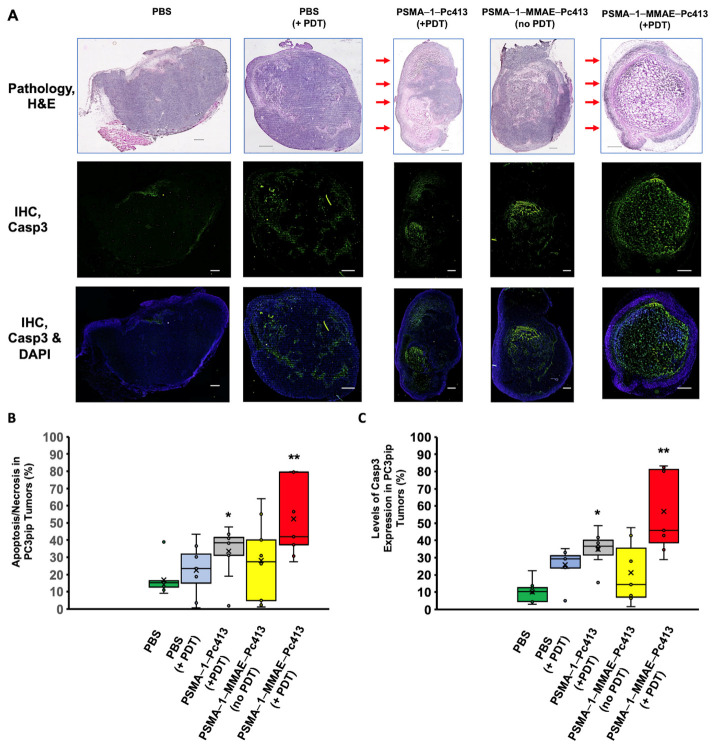
Treatment caused apoptosis in PC3pip tumor. Mice received 100 nmol/kg of PSMA-1-MMAE-Pc413 and PDT was performed at 24 h post-injection. Mice were sacrificed 3 days after PDT and tumors were extracted for histological analysis. (**A**) H&E staining and Caspase 3 assay of treated tumors. Red arrows indicate the area that was directly exposed to light. Scale bar = 500 μm. (**B**) Quantification of tumor necrosis/apoptosis from H&E staining. (**C**) Quantification of Caspase-3 fluorescent signal intensity in tissues. In (**B**,**C**), box—middle 50% of data points, line—median, cross—average level, whiskers—range of the data, excluding any outliers in (**B**,**C**). *: *p* < 0.001 PSMA-1-Pc413 (+PDT) to PBS; **: *p* < 0.05 PSMA-1-MMAE-Pc413 (+PDT) to all other groups.

To further compare the tumor damage after treatments, a subset of PC3pip tumors from different treatment groups was collected 3 days after a single treatment (one dose) and analyzed histologically (Figure 6). H&E staining showed that control tumors that received PBS had 16.9 ± 10.9% necrotic/apoptotic damage while tumors having received PBS and treated with light only showed 23.4 ± 14.8% damage (Figure 6A,B). The treatment of the tumors with PSMA-1-Pc413 with PDT significantly increased tumor necrotic/apoptotic damage to 33.6 ± 20.4%, which was mainly found in the area directly exposed to light. The treatment of tumors with PSMA-1-MMAE-Pc413 without PDT caused 28.0 ± 16.3% damage to the tumor but the damage was mainly found in the center of the tumor. In contrast to these four groups, the combination treatment of PSMA-1-MMAE-Pc413 with PDT significantly increased the level of necrosis/apoptosis to 52.4 ± 22.3% in the tumor, and damage was distributed throughout the tumor. The further staining of tumor sections for Caspase 3 concurred with H&E staining results, with most apoptosis found in combination treatment groups (Figure 6A,C). These results suggested that the combination treatment of PDT and MMAE is the most efficacious.

### 2.6. Toxicity Studies of PSMA-1-MMAE-Pc413 in Mice

Toxicity studies in mice were performed to determine the target organ toxicity, maximum tolerated dose (MTD), and no-observed-adverse-effect level (NOAEL) of PSMA-1-MMAE-Pc413. Based on a single-dose Phase A study, in which all mice survived the dose at 5.0 mg/kg, doses of 0.5, 1.5, and 5.0 mg/kg were used in the three-weekly dose Phase B study. No drug-related mortalities were observed. The mean group body weights were significantly decreased by day 16 only in the high-dose group (*p* < 0.001) and no significant changes in body weights were seen by the end of the recovery period (day 30). Clinical chemistry data and histopathology evaluation are summarized in Tables S5 and S6. In summary, the 0.5 mg/kg/week (equal to 136 nmol/kg) dose administered intravenously (i.v.) for 3 consecutive weeks should be considered the NOAEL under the conditions of this study. The dose of 1.5 mg/kg/week (equal to 408 nmol/kg) should be considered minimally toxic and the dose of 5 mg/kg/week (equal to 1360 nmol/kg) can be considered the MTD following three injections once a week.

## 3. Discussion

High-risk localized prostate cancer exhibits significant variability in its characteristics, impacting treatment response and overall clinical outcomes. The diversity of prostate cancer cells calls for employing combination therapies with diverse mechanisms over singular therapeutic approaches. Integrating multiple drugs poses complexities, such as differences in pharmacokinetics, affecting drug delivery to tumors and causing unintended effects in other bodily tissues, limiting the utility of anticancer drugs due to adverse reactions. To overcome this, we have developed a multifaceted molecule, PSMA-1-MMAE-Pc413, which is uniquely tailored to target the PSMA biomarker, and combines Pc413-based PDT and MMAE-based chemotherapy in one molecule. PSMA-1-MMAE-Pc413 showed selective binding to PSMA-positive PC3pip cells (Figure 3A,B). Similar to PSMA-1-Pc413 [37], after entering cells, it is located in the perinuclear region. This selective uptake resulted in the enhanced cytotoxicity of PSMA-1-MMAE-Pc413 to PC3pip cells (Figure 3C and Table S1). It was noticed that light irradiation significantly increased the cytotoxicity of PSMA-1-MMAE-Pc413 and this enhancement is synergistic as indicated by the CDI value (0.001 < 1). In vivo fluorescence imaging studies again demonstrated selective uptake in PC3pip tumors (1.6-fold higher than in PC3flu tumors) with a peak accumulation time of 24 h post-injection. The in vivo treatment of PC3pip tumors showed that a combination of PDT and MMAE (PSMA-1-MMAE-Pc413 + PDT) significantly delayed tumor growth rates and increased animal survival times when compared to either chemotherapy alone (PSMA-1-MMAE-Pc413 with no PDT) or PDT alone (PSMA-1-Pc413 + PDT); see Figure 5 and Tables S2–S4. Toxicity studies found that the NOAEL of PSMA-1-MMAE-Pc413 was 0.5 mg/kg (equal to 136 nmol/kg), which is higher than the dose (100 nmol/kg) used in treating PC3pip cells; therefore, no severe adverse effects are expected from the treatment. Consequently, no body weight loss was observed during the treatment (Figure 5D).

Interestingly, during the treatment, it was noticed that subsequent fluorescence signal measurements of PC3pip tumors treated with PSMA-1-MMAE-Pc413 + PDT were significantly increased after the first PDT, and the signal was also higher than those measured for the PSMA-1-MMAE-Pc413 without PDT and PSMA-1-Pc413 + PDT groups. These observations indicated an increased drug uptake in the PC3pip tumors treated with PSMA-1-MMAE-Pc413 + PDT. One of the mechanisms of PDT-based anticancer treatment is the destruction of tumor vasculature by ROS [23,42,43,44]. Further, it is possible that the added efficacy of MMAE also impacts vascular integrity, improving the uptake of PSMA-1-MMAE-Pc413 compared to agents that only deliver a single form of therapy [45]. This damage can increase vascular leakiness and permeability, resulting in increased drug uptake in tumors and a subsequently improved antitumor efficacy [46,47,48]. Our results indicate that PSMA-1-MMAE-Pc413 traverses through the cancer vasculature and that PDT activity from the Pc413 portion of the molecule allows the destruction of these tumor-associated structures upon the application of NIR light, leading to more drug accumulation in the tumor after the second injection as indicated with fluorescence imaging (Figure 5A). This increased drug uptake needs to be further confirmed through the extraction of the drug from tumor tissue.

Although PDT has been used as a therapeutic modality for cancer, its use is confined by limited light penetration, which is not beyond a few millimeters [49], making it difficult to treat a large tumor mass. Our histological studies of PSMA-1-Pc413-based PDT showed that the damage of PDT is located on the superficial area of the tumor, which was directly exposed to light (Figure 6). In contrast, chemotherapy alone (PSMA-1-MMAE-Pc413 with no PDT) mainly destroyed the center of the tumor. Significantly, when the tumor was treated with the combination of PDT and chemotherapy (PSMA-1-MMAE-Pc413 + PDT), necrotic/apoptotic destruction was found throughout the tumor. These results showed histological evidence that combination treatment is more effective than single treatments.

We have previously reported a multifunctional molecule, PSMA-1-MMAE-IR700 [35], which also demonstrated enhanced antitumor activity when used in combination treatment. Comparing PSMA-1-MMAE-IR700 and PSMA-1-MMAE-Pc413, the only difference between the two molecules is the photosensitizer. This change greatly impacted the pharmacokinetics of the final compound. PSMA-1-MMAE-Pc413 had a blood half-life (t_1/2_) of 7.55 ± 0.91 h (Figure S1), while that of PSMA-1-MMAE-IR700 was 0.27 ± 0.05 h (Figure S3). There have been many reports that the fluorophores attached to the molecule will affect the pharmacokinetics of the molecule dramatically [37,39,50]. Compared to IR700, Pc413 lacks the six sulfate groups and is more hydrophobic, resulting in longer circulation time in the body. In reflection of this longer circulation time, PSMA-1-MMAE-Pc413 was dosed every 7 days instead of every 4 days, as was PSMA-1-MMAE-IR700. Pc413 has a higher singlet oxygen quantum yield (0.43) than that of IR700 (0.3) [28], which suggests that Pc413 is a more powerful photosensitizer than IR700. In fact, it only required three doses of PSMA-1-MMAE-Pc413 PDT to achieve a 60% cure, while PSMA-1-MMAE-IR700 required five doses to achieve this treatment efficacy [35]. This dose frequency can be further reduced by applying PDT only once 24 h after the first injection of PSMA-1-MMAE-Pc413 (Figure S4). Animals in this treatment group had a mean survival of 86.8 days while the PBS group had a mean survival of 37.2 days (*p* < 0.0001). This reduced dose frequency may improve the patients’ quality of life and reduce the cost of the treatment. Although our fluorescence-based studies provide some information on the pharmacokinetic behavior of PSMA-1-MMAE-Pc413, we are aware that MMAE may be released in the body as it is conjugated through a cathepsin-cleavable linker; therefore, fluorescence cannot fully represent the behavior of the whole intact molecule. A detailed pharmacokinetic study of PSMA-1-MMAE-Pc413 is still needed to fully understand the interaction of PSMA-1-MMAE-Pc413 within the body, which can usually be conducted via liquid chromatography–mass spectrometry (LC-MS) to quantitatively and qualitatively study the metabolism of the drug.

In clinical applications, PSMA-1-MMAE-Pc413 could be used to treat patients without surgery. Alternatively, the fluorescence of Pc413 makes it possible to perform fluorescence image-guided surgery (FIGS). We have previously demonstrated that PSMA-1-Pc413 can provide effective adjuvant PDT after the image-guided surgery of prostate cancer, resulting in surgical cures [29]. Compared to PSMA-1-Pc413, PSMA-1-MMAE-Pc413 and PSMA-1-MMAE-IR700 can contribute additional chemotherapy to the surgical bed after FIGS and PDT. However, PSMA-1-MMAE-IR700 demonstrated peak tumor accumulation 1 h post-injection followed by a subsequent active clearance [35]. The efficacy of PDT after surgery might diminish as the agent continues to clear from the system. In contrast, PSMA-1-MMAE-Pc413 had a peak accumulation time at 24 h post-injection and was cleared relatively slowly from the tumor. This distinctive biodistribution profile will allow for a greater time frame to perform surgery and PDT; therefore, PSMA-1-MMAE-Pc413 might be better suited for this FIGS and adjuvant PDT. Additionally, Pc413 has a higher fluorescence quantum yield (0.4) than IR700 (0.24) [28]; thus, a higher sensitivity is expected during FIGS. Studies investigating the efficacy of PSMA-1-MMAE-Pc413 for FIGS/PDT are currently underway.

Recently, the stimulation/de-repression of the immune system, i.e., immune therapy, has been employed to treat cancer [51]. In this regard, PDT is resurfacing as a potential therapy for local and systemic disease, as the local application of PDT-generated ROS can result in immunogenic cell death (ICD), solicits a dramatic immune response, and promotes immunologic memory that protects animals against tumor rechallenge [52,53,54]. Further, an antibody–drug conjugate (ADC), FDA-approved brentuximab vedotin, which utilizes MMAE as its chemotherapeutic payload, has been demonstrated to elicit the induction of the classic hallmarks of ICD and protected animals from tumor rechallenge in preclinical studies. We are currently exploring the immune response after PSMA-1-MMAE-Pc413 or PSMA-1-Pc413 therapy in immune-competent animal models of prostate cancer.

## 4. Material and Methods

### 4.1. Synthesis of PSMA-1-MMAE-Pc413

Succinimidyl- *trans*-4-(*N*-maleimidylmethyl)cyclohexane-1-carboxylate (SMCC, 2 mg, 6.0 μmol) (Sigma) was dissolved in 0.5 mL of dimethylformamide (DMF). The pH of the solution was adjusted to 8.0 using triethylamine (TEA). Pc413 (5 mg, 5.9 μmol) was dissolved in 1 mL of a mixture of DMF/methanol/chloroform (1:1:1). The two solutions were mixed and stirred at room temperature in the dark for 1 h, then the reaction mixture was added directly to PSMA-1-VcMMAE [35,38] (10 mg, 3.8 μmol) in DMF (1 mL). The reaction was carried out at room temperature and the final product was purified via semi-preparative high-performance liquid chromatography (HPLC). Appearance: blue powder. Yield: 8 mg, 57%. Purity: 96.5% (Figure S5). Melting point: 106 °C. Mass spectrum (MS): C_179_H_257_N_33_O_42_S_2_Si_3_, calculated 3686; found: 3685 [M-1]; 1842 [M-2]/2 (Figure 2B).

IR (ν_max_, cm^−1^): 3293, 3060, 2913, 2319 cm^−1^, 1698, 1637, 1542, 1449 1399, 1335, 1250, 1122, 1080, 910, and 741 cm^−1^ (Figure S6).

^1^H NMR (500 MHz, DMSO-*d_6_*): δ (ppm) 10.05 (s, 1H), 9.69–9.67 (m, 8H), 8.49–8.47 (m, 8H), 8.36–8.18 (m, 4H), 8.06–7.85 (m, 4H), 7.69–7.59 (m, 4H), 7.43 (s, 1H) 7.24–7.34 (m, 6H), 7.19–7.15 (m, 1H), 6.92 (s, 1H), 6.39 (d, 0.5H), 6.27 (s, 0.5H), 6.05 (s, 0.5H), 5.44 (s, 1H), 5.04 (m, 1H), 4.49 (d, 1H), 4.43 (d, 1H), 4.38 (m, 2H), 4.29–4.24 (m, 1H), 4.21–4.17 (m, 4H), 4.15–4.10 (m, 1H), 4.04–3.92 (m, 5H), 3.78 (d, 1H), 3.24–3.12 (m, 30H), 3.08–2.95 (m, 16H), 2.88–2.84 (m, 5H), 2.28–2.16 (m, 9H), 2.14–2.06 (m, 10H), 2.01–1.94 (m, 4H), 1.91–1.81 (m, 16H), 1.71–1.63 (m, 10H), 1.55 (d, 4H), 1.50–1.42 (m, 18H) 1.39–1.32 (m, 9H), 1.29–1.07 (m, 16H), 1.05–1.00 (m, 5H), 0.88–0.81 (m, 21H), 0.79–0.73 (m, 9H), 0.71–0.68 (m, 2H), −1.08–1.17 (m, 2H), −1.24–1.30 (m, 2H), −2.16–2.20 (m, 2H), and −2.27–2.3 (m, 2H), −2.92–2.95 (m, 12H) (Figure S7).

^13^C NMR (126 MHz, DMSO-*d6*): δ (ppm) 176.64, 176.53, 176.17, 175.01, 174.91, 174.78, 174.66, 174.63, 174.10, 172.25, 172.11, 171.75, 171.46, 171.37, 171.24, 170.56, 170.02, 169.74, 169.26, 168.59, 168.23, 162.32, 158.82, 157.79, 157.73, 157.23, 154.87, 153.88, 152.0, 151.90, 149.90, 149.48, 148.38, 148.10, 144.97, 143.52, 134.96, 131.63, 128.01, 127.66, 127.60, 126.59, 126.31, 126.27, 123.44, 118.79, 107.0, 100.79, 96.79, 85.19, 81.45, 74.44, 72.28, 65.79, 60.81, 60.28, 60.14, 58.53, 58.05, 58.49, 57.59, 56.96, 56.22, 55.55, 53.97, 53.15, 52.96, 52.92, 52.71, 52.52, 52.49, 52.38, 52.35, 52.27, 49.62, 47.11, 45.43, 44.44, 44.00, 43.78, 43.65, 43.57, 43.06, 42.49, 41.26, 38.60, 38.07, 37.98, 36.93, 35.68, 35.49, 35.23, 35.01, 34.96, 34.93, 34.83, 32.31, 31.99, 31.81, 31.53, 31.37, 31.22, 31.05, 30.26, 29.47, 29.25, 29.17, 29.12, 28.71, 28.48, 28.41, 27.03, 26.99, 26.66, 25.93, 25.89, 25.70, 25.20, 24.83, 24.80, 24.74, 24.16, 22.97, 22.71, 21.17, 20.49, 19.10, 18.90, 18.78, 18.75, 18.68, 18.62, 18.58, 18.38, 18.12, 17.91, 15.63, 15.31, 15.19, 15.12, 14.84, 14.49, 12.37, 10.75, 10.49, 10.25, 10.15, −3.52, and −3.80 (Figure S8).

### 4.2. Cell Culture and Maintenance

PSMA-positive PC3pip cells and PSMA-negative PC3flu cells were originally obtained from Dr. Michel Sadelain in 2000 (Laboratory of Gene Transfer and Gene Expression, Gene Transfer and Somatic Cell Engineering Facility, Memorial-Sloan Kettering Cancer Center, New York, NY, USA). Cells were last checked for PSMA expression using Western Blot analysis and flow sorting in February 2023; no genetic authentication was performed. Cells were maintained in RPMI-1640 medium (Invitrogen Life Technology, Carlsbad, CA, USA) with 10% FBS at 37 °C and 5% CO_2_ under a humidified atmosphere. Cells were all tested for microplasma in February 2023.

### 4.3. In Vitro Uptake Studies

For cellular uptake studies, PC3pip or PC3flu cells (5 × 10^5^ cells) were incubated with 50 nM of PSMA-1-MMAE-Pc413 at various times. After incubation, cells were washed 3 times with cold phosphate-buffered saline (PBS), re-suspended in PBS, and transferred to 96-well black plates. Fluorescence was measured using a Tecan Microplate Reader (Tecan Life Sciences, Männedorf, Switzerland) using excitation at 610 nm and emission at 672 nm. Blocking experiments were performed via the co-incubation of the cells with 50 nM of PSMA-1-MMAE-Pc413 and 500 nM of ZJ24 [37,39]. The uptake of PSMA-1-MMAE-Pc413 was also checked using fluorescence images. Cells were plated on coverslips at about 70% confluency. Twenty-four hours later, cells were incubated with 50 nM of PSMA-1-MMAE-Pc413 for 4 h. After incubation, cells were washed three times with PBS, fixed with 4% paraformaldehyde, counterstained with 2-(4-amidinophenyl)-6-indolecarbamidine dihydrochloride (DAPI), mounted with Fluor-Mount aqueous mounting solution, and observed. Images were taken using a Leica DM4000B fluorescence microscope (Leica Biosystems, Buffalo Gove, IL, USA) at 40 × magnification. Blocking experiments were performed via the co-incubation of cells with 50 nM of PSMA-1-MMAE-Pc413 and 500 nM of ZJ24. Studies were performed in triplicate.

### 4.4. In Vitro Cytotoxicity Assay

For the cytotoxicity assay, PC3pip and PC3flu cells were plated at 3,000 cells/100 μL/well in 96-well plates. Twenty-four hours later, different concentrations of PSMA-1-MMAE-Pc413 or PSMA-1-Pc413 were added. After incubation at 37 °C in the dark for 24 h, cells were washed 3 times with RPMI 1640 medium, irradiated with light using irradiance at 8.3 mW/cm^2^ and irradiant exposure at 5.0 J/cm^2^ light (>500 nm, Apollo Horizon Projector, Acco Brands, Lake Zurich, IL, USA) [37], and put back into the incubator. Cell proliferation was determined 24 h later via CellTiter 96 Aqueous One Solution Cell Proliferation Assay (Promega, Madison, MI, USA) using absorbance at 490 nm. The concentration required to inhibit 50% of the cell proliferation (IC_50_) was calculated using Prism Graph Pad 10. The coefficient drug index (CDI) is calculated as CDI = IC_50_ (AB)/[IC_50_(A) × IC_50_ (B)], where AB is the combination treatment, and A and B are single treatments. CDI < 1 is considered synergistic [35,55].

### 4.5. In Vivo Fluorescence Imaging Studies

Animal experiments were approved by the University Institutional Animal Care and Use Committee (IACUC), Protocol Number: 2015-0033. Six- to eight-week-old male athymic mice, obtained from Jackson Laboratory, were subcutaneously implanted with 1 × 10^6^ PC3pip cells on the right flank and 1 × 10^6^ PC3flu cells on the left flank of the mouse in 100 µL of a 1:1 PBS/Matrigel mixture. These tumors were allowed to grow to around 100 mm^3^ (tumor volume = length × width^2^/2) and were injected with 100 nmol/kg of PSMA-1-MMAE-Pc413 via tail vein injections. Mice were imaged at various timepoints using the Maestro In Vivo Imaging System (Perkin Elmer, Waltham, MA, USA) with the yellow filter set (excitation 575–605 nm, emission 645 nm long pass). At 72 h post-injection, the mice were sacrificed and tumors and organs were extracted and once again imaged. Average fluorescence signals on tumors or organs were quantified, which is the total fluorescence signal (in scaled counts/s or counts) normalized with the tumor area by drawing regions of interest (ROIs) on selected areas using Maestro 2.0 software.

### 4.6. In Vivo Survival Study

Male athymic nude mice were implanted subcutaneously with 1 × 10^6^ of PC3pip cells in 100 μL of PBS/Matrigel mixture (1:1) in the right flanks. When tumor size reached approximately 50–80 mm^3^, mice received 100 nmol/kg of PSMA-1-MMAE-Pc413 or PSMA-1-Pc413 through tail vein injection based on the treatment plan or sterile PBS as a negative control. Mice received drugs every 7 days with a total of 3 doses. For those groups treated with PDT, 150 J/cm^2^ of laser irradiation using a 672 nm laser (Modulight, Tampere, Finland) was administered (irradiance of 300 mW/cm^2^) 24 h after each injection. The mice were imaged before each injection, 24 h post-injection, and immediately after PDT using the Maestro In Vivo Imaging System (Perkin Elmer)—the yellow filter was used to visualize the tumor signals with a 575–605 nm excitation filter and a long-pass 645 nm emission filter. Animals were weighed and tumor size was measured every other day for 90 days using calipers. When assessing information, cures were defined as no tumor present at the end of the 90-day study. When tumors became too large (>2 cm in diameter) or animals were moribund, they were euthanized. Five mice were used in each group.

### 4.7. Histology and Immunofluorescent Analysis of Tissue Samples

Mice with PC3pip tumors received 100 nmol/kg of drug through tail vein injection and were imaged 24 h post-injection. After imaging, mice in + PDT groups were irradiated with 150 J/cm^2^ of 672 nm light (Modulight). Mice were sacrificed 3 days later, and tumor xenograft samples were excised and snap-frozen in optimum cutting temperature compound (OCT) for cryo-sectioning (Leica CM3050S). Sections, 12 μm thick, were serially collected directly onto slides and stored at −80 °C for further processing. For immune-histochemical analysis (IHC), the slides were warmed to room temperature (RT) for 10 min, fixed with 10% buffered formalin, blocked in blocking buffer (5% normal goat serum/0.3% Triton X-100 in 1X PBS) for 1 h at RT, and incubated overnight in primary antibody followed by three 5 min washes. The presence of apoptosis in the tumor was evaluated using rabbit anti-Cleaved Caspase-3 antibody (#700182, Cell Signal Tech, Danvers, MA, USA) at a 1:400 dilution. After washing, the slides were treated with the secondary ready-to-use goat anti-rabbit polyclonal antibody labeled using Alexa Fluor-488 (Invitrogen, Inc., Waltham, MA, USA) for 20 min at RT followed by double washing with PBS for 5 min. Tissue nuclei were contrasted with Fluoro-Gel-II containing DAPI (Electron Microscopy Sciences, Hatfield, PA, USA). Additionally, adjacent slides were stained for hematoxylin and eosin (H&E) according to standard procedures. Fluorescent and H&E images were viewed with a Keyence BZ-X810 fluorescent microscope (Osaka, Japan), digitized (Keyence BZ-H4XD module), and analyzed with Keyence-BZ-X800 analyzer software.

### 4.8. In Vivo Toxicity Studies

The toxicity study was conducted by the Center for Biomedical Testing (Chicago, IL, USA). Male CD-1 mice aged 6–8 weeks old were used in the study. The studies included a single-dose Phase A study and a 3-weekly dose Phase B study. For the Phase A study, 10 CD-1 mice were assigned to the control or 5 single-dose groups in the range of 5–100 mg/kg of PSMA-1-MMAE-Pc413 to select the doses for the weekly 3-dose study. In Phase A, all animals in the 50 mg/kg and above dose groups died following a single intravenous (i.v.) injection. The animals in the 12.5 mg/kg and 5 mg/kg survived the single i.v. injection of PSMA-1-MMAE-Pc413. Two animals in the 5 mg/kg dose group were dosed with 2 additional weekly injections and also survived. This dose was. Therefore. selected as a high dose for the 3 weekly injections in Phase B of the study. In Phase B, 40 (10/group) male CD-1 mice were randomly divided (*n* = 10/group) into 4 groups (one vehicle control and 3 drug-dose groups, low, mid, and high at 0.5, 1.5, and 5 mg/kg/week, respectively). PSMA-1-MMAE-Pc413 formulation in saline (or sterile saline vehicle in the control group) was administered intravenously to animals weekly over 3 consecutive weeks based on their groups. Five animals per dose group were sacrificed on the next day after the last injection (day 16) and the remaining animals were sacrificed on day 30 (two weeks after the last injection, i.e., recovery group animals). A thorough necropsy was performed on all animals and the heart, lungs, spleen, liver, kidneys, and salivary glands were collected in 10% neutral buffered formalin. Clinical chemistry, hematology studies, and histopathological evaluation were performed on all animals.

### 4.9. Statistical Analysis

Student’s *t*-test was used to compare differences between groups. For survival studies, SAS9.4 statistical software was used for survival analysis with Kaplan–Meier survival curves using Log-rank (Mantel–Cox) test. A *p* value < 0.05 is considered statistically significant between groups.

## 5. Conclusions

In summary, the synthesis of PSMA-1-MMAE-Pc413 represents a significant advancement in the treatment of prostate cancer. By combining imaging, chemotherapy, and PDT into a single theranostic agent, PSMA-1-MMAE-Pc413 offers a synergistic therapeutic effect that surpasses the efficacy of each treatment modality alone and offers more efficacy than PSMA-1-MMAE-IR700, another such PDT/chemotherapy combination. The findings from this study suggest that PSMA-1-MMAE-Pc413 has strong potential for clinical application, providing a promising new approach for enhancing the treatment of prostate cancer. Furthermore, PSMA-1-MMAE-Pc413 is specifically designed to target PSMA. Although PSMA is expressed in more than 90% of prostate cancer patients, there are patients with no or low PSMA expression. PSMA-targeted PET scans may be required to select patients for this treatment [17,19]. To explore the full therapeutic potential of PSMA-1-MMAE-Pc413, future work will include the use of PSMA-1-MMAE-Pc413 for image-guided surgery followed by PDT and investigating immune response initiated by PSMA-1-MMAE-Pc413 + PDT.

## Data Availability

All data generated or analyzed in this study are included in this article. The raw data supporting the conclusions of this article will be made available by the authors upon request.

## Supplementary Materials

The following supporting information can be downloaded at: https://www.mdpi.com/article/10.3390/ijms25137086/s1.
