# Supplementary material for: A Synergistic Strategy Combining Chemotherapy and Photodynamic Therapy to Eradicate Prostate Cancer
Source: Int J Mol Sci. 2024 Jun 28;25(13):7086. doi: 10.3390/ijms25137086 (PMC11241360; doi:10.3390/ijms25137086)
Supplement: Supplementary file 1 [file ijms-25-07086-s001.zip › ijms-3060792-supplementary.pdf]

Table S1: Summary of IC_{50} values in nM. Values are mean ± SD of six replicates. (NA: not available.)

	Cell line	IC_{50} (nM) (0 J/cm^2)	IC_{50} (nM) (5 J/cm^2)
**PSMA-1-Pc413**	PC3pip	NA	164.9±33.1
**PSMA-1-Pc413**	PC3flu	NA	308.2±46.5
**PSMA-1-MMAE-Pc413**	PC3pip	12.6±1.1	2.2±0.4
**PSMA-1-MMAE-Pc413**	PC3flu	46.6±3.2	14.7±0.5

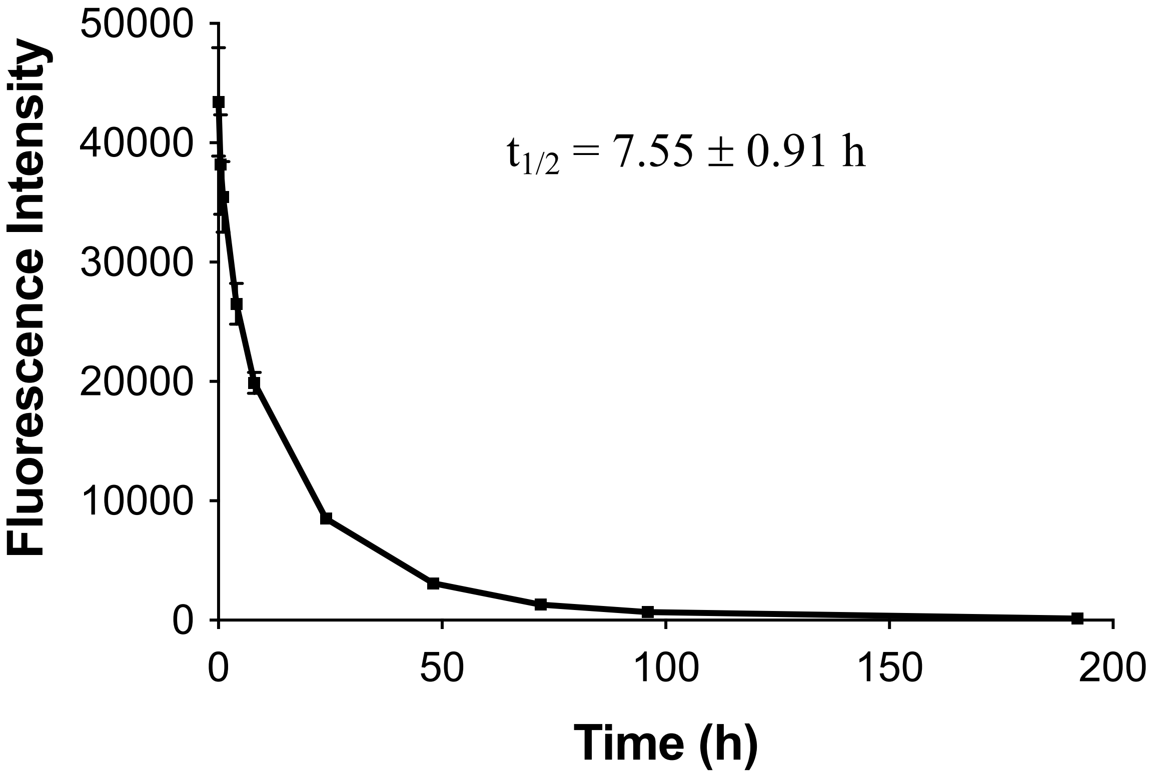

Figure S1. Pharmacokinetic studies of PSMA-1-MMAE-Pc413 in male nude mice. Mice received 100 nmol/kg of PSMA-1-MMAE-Pc413 through tail vein injection. Blood was taken at different time points. Fluorescence in the blood plasma was then measured (excitation 630 nm, emission at 672 nm). Values are mean±SD of 5 animals.

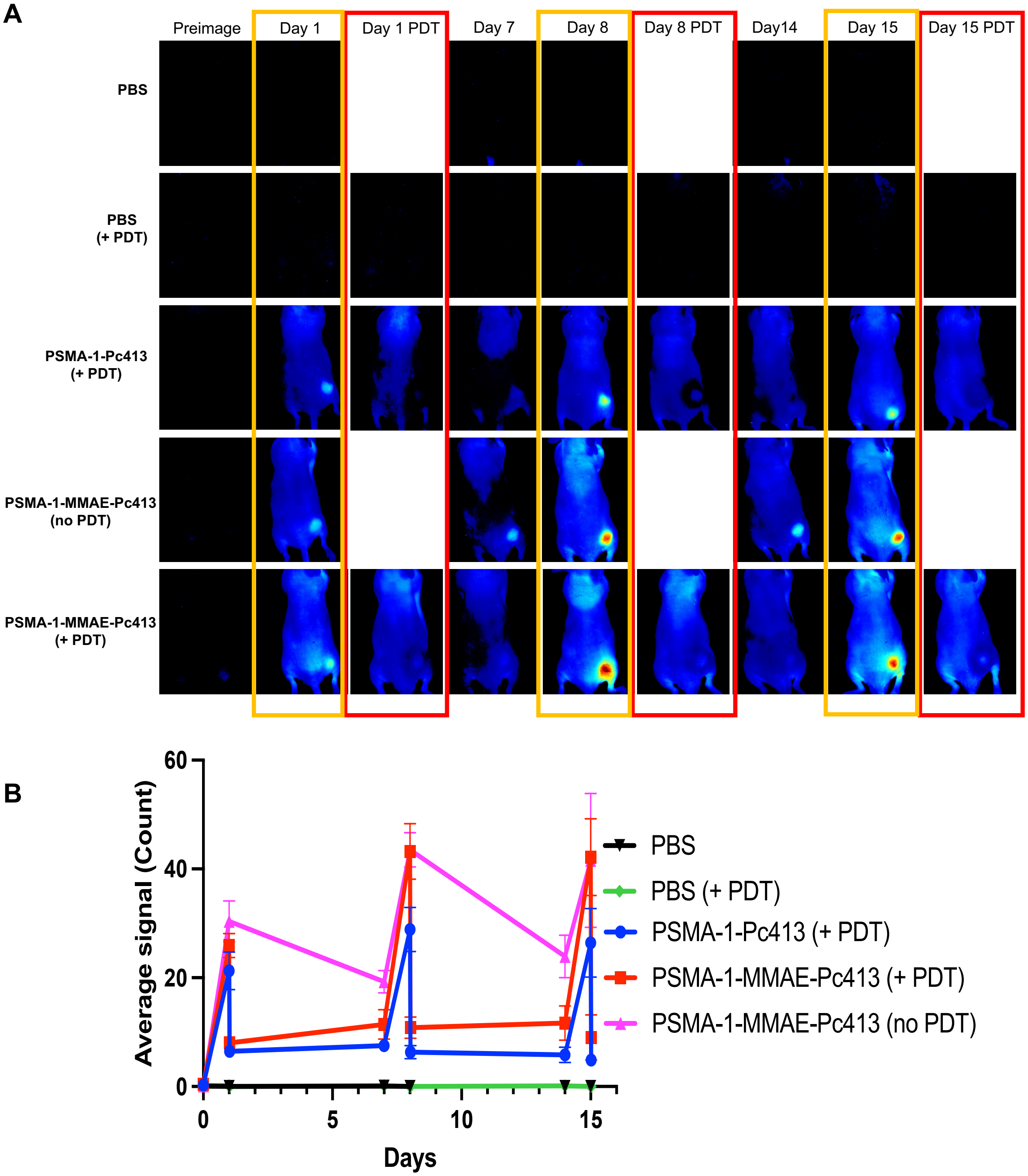

Figure S2. Maestro imaging of mice in the treatment groups. (**A**) Fluorescence images of mice in different treatment groups. Drugs were given on day 0, day 7 and day 14. Mice were imaged 24 hours after injection on day 1, day 8 and day 15 as indicated by orange boxes. Right after imaging, PDT was applied. Mice were imaged again after PDT as indicated in red boxes. Pictures are representative images of 5 mice. (**B**) Quantification of average fluorescence signals on PC3pip tumors. Values are mean± SD of 5 animals.

Table S2. Summary of p values of tumor growth curves.

Mann Whitney t- test	*PBS*	*PBS (+PDT)*	*PSMA-1-Pc413 (+PDT)*	*PSMA-1-MMAE-Pc413 (no PDT)*	*PSMA-1-MMAE-Pc413 (+PDT)*
PBS	X	>0.9999	0.0457	0.0058	<0.0001
PBS (+PDT)	>0.9999	X	0.0337	0.0044	<0.0001
PSMA-1-Pc413 (+PDT)	0.0457	0.0337	X	0.0999	<0.0001
PSMA-1-MMAE-Pc413 (no PDT)	0.0058	0.0044	0.0999	X	0.0487
PSMA-1-MMAE-Pc413 (+PDT)	<0.0001	<0.0001	<0.0001	0.0487	X

Table S3. Summary of p values of Kaplan-Meier survival curves.

Mann Whitney t- test	*PBS*	*PBS (+PDT)*	*PSMA-1-Pc413 (+PDT)*	*PSMA-1-MMAE-Pc413 (no PDT)*	*PSMA-1-MMAE-Pc413 (+PDT)*
PBS	X	0.9206	0.1905	0.0079	<0.0001
PBS (+PDT)	0.9206	X	0.1667	0.0079	0.0001
PSMA-1-Pc413 (+PDT)	0.1905	0.1667	X	0.2143	0.0034
PSMA-1-MMAE-Pc413 (no PDT)	0.0079	0.0079	0.2143	X	0.0469
PSMA-1-MMAE-Pc413 (+PDT)	<0.0001	0.0001	0.0034	0.0469	X

Table S4. Summary of mean survival time from Kaplan-Meier survival curves.

Mean survival time	*PBS*	*PBS (+PDT)*	*PSMA-1-Pc413 (+PDT)*	*PSMA-1-MMAE-Pc413 (no PDT)*	*PSMA-1-MMAE-Pc413 (+PDT)*
Days	37	38	48	57	90

Table S5. Summary of clinical chemistry and hematology data that did not completely recover on day 16 and day 30.

**Weekly Dose (mg/kg):**	**0 (control)**	**0.5 mg/kg/week**	**1.5 mg/kg/week**	**5 mg/kg/wee k**
**Sex: Number of Animals:**	**M:** 5	**M:** 5	**M:** 5	**M:** 5
**Clinical Chemistry (Phase B)**				
ALT (U/L) (Day 30)	29.8	38.33	65.4	72.2*
AST (U/L) (Day 30)	74.4	102.67	197.6	222**
LDH (U/L) (Day 30)	1182.6	1358	2023.6*	1827.0
CK (U/L) (Day 30)	1085.8	1607.67	4108.2*	2312*
Albumin (Day 16)	2.8	2.8	3.02*	2.8
TBILI, mg/dL (Day 30)	0.3	0.27	0.16*	0.12*
BIL, unconj. (mg/dL) (Day 30)	0.28	0.20	0.16*	0.12**
Glucose, mg/dL (Day 16)	161.75	152.0	120.8	89.2**
Glucose, mg/dL (Day 30)	260.0	269.33	178.6*	158.8**
Triglycerides (mg/dL) (Day 30)	196.0	243.33	190.0	94.2**
Ca, mg/dL (Day 16)	8.68	9.08	8.76	9.72*
Chloride (mMol/L) (Day 30)	104.75	104.33	105.8	111.2**
Potassium (mMol/L) (Day 30)	4.48	4.8	4.9	5.18**
Sodium (mMol/L) Day 30	150.25	149.0	153.4*	154.2*
**Hematology**				
WBC, (K/uL) (Day 16)	3.148	3.03	6.12	10.43**
WBC, (K/uL) (Day 30)	5.91	6.35	8.40	8.97*
NEU (K/uL) (Day 16)	0.95	0.93	2.56	6.74*
NEU (K/uL) (Day 30)	1.78	1.65	3.85	5.02**
LYM (K/uL) Day 16	1.77	1.59	2.9*	2.75
EO (K/uL) Day 16	0.006	0.005	0.02*	0.23
EO (K/uL) Day 30	0.01	0.04	0.024	0.03*
MO (K/uL) Day 30	0.42	0.69	0.99*	1.20
RBC (M/uL) Day 16	9.23	8.97	9.06	8.38*
Hb (g/dL) Day 16	14.76	14.75	15.06	13.4**
Hb (g/dL) Day 30	15.44	14.47	14.38	13.92*
RDW (%) Day 16	18.84	18.80	20.42**	19.86*
RDW (%) Day 30	18.46	17.6	20.52*	21.18*
MPV (fL) Day16	4.82	5.1	5.46	5.46*
PLT (K/uL) Day 30	990.6	881.67	1291.6	1355.6*
**Body Weights Change (%)**				
Day 1 to Day 16	2.34	1.74	3.38	-8.31***
Day 16 to Day 30	7.95	3.64	7.26	10.03
**Clinical Signs#**				
Green/Bluish Bedding (Urine)	-	-	3-4 (6-10)	8 (7-10)

Alopecia, an open wound on head/shoulder/neck (Day 1-16) N=10	-	-	-	1 (10)<br>3 (11)<br>8 (12)<br>9 (13-16)
Alopecia, an open wound on the head/shoulder/neck (Day 17-30) N=5			1/5 (17-18)<br>4/5 (20-30)	5/5 (17-30)
**Gross Necropsy Observations** (Day 16)	-	-	-	Kidney and spleen pale color
**Microscopic Observations^**<br>**Liver:** Small inflammatory cell foci (Day 16)<br>**Spleen:** Depletion of red pulp (Day 16)<br>Spleen: Hypercellularity of red pulp (Day 30)	1/5 (0.2)<br>1/5 (0.2)<br>-	-<br>3/5 (0.8)<br>-	-<br>3/5 (0.6)<br>2/5 (0.4)	2/5 (0.8)<br>5/5 (2.8)**<br>3/5 (0.6)

- No noteworthy findings.

* Statistically significant difference from the vehicle control group ($p<0.05$)
** Statistically significant difference from the vehicle control group ($p<0.01$)
*** Statistically significant difference from the vehicle control group ($p<0.001$)

Data represented as no. of animals with symptoms/total number of animals in the group. The number in parenthesis indicates the days on which observations were made.

^ Data represented as no. of animals with lesion/total number of animals in the group. The number in parenthesis indicates the mean group severity score.

Table S6. Summary of histopathology findings in animals (necropsy on day 16). (Group 1: PBS, group 2: 0.5 mg/kg, Group 3: 1.5 mg/kg and Group 4: 5.0 mg/kg).

	Group 1					Group 2					Group 3					Group 4				
Animal	287L	272L	272N	286B	273R	276R	276N	276L	277R	277L	278R	288N	278L	278N	279R	285L	281N	281L	281R	282R
Tissue & Lesions																				
Spleen																				
Depletion of red pulp	0	0	1	0	0	0	0	1	1	2	1	0	1	0	1	3	3	3	2	3
Liver																				
No abnormalities detected		p	p	p	p								p			p		p	p	
Small inflammatory cell foci	1					1											1.5			2.5
Microvesicular fat accumulation - midzonal-centrilobular							2.5		2											
Glycogen-like accumulation - midzonal-centrilobular	1							1.5				2			2					
Diffuse centrilobular cell swelling											1									
Areas of centrilobular cell swelling										1										
Small areas of sub-acute necrosis	four																			
Small hemorrhage														p						
Small area of hydropic degeneration														p						
Small area of venous congestion										p										
Kidneys																				
No abnormalities detected	p	p	p		p	p	p	p	p	p	p	p	p	p		p	p	p	p	p
Small focus of tubular nephrosis				p																
Couple of small hemorrhages at the capsule															p					
Heart																				
No abnormalities detected	p	p	p	p	p	p	p	p	p	p	p	p	p	p	p	p	p	p	p	p
Lungs																				
No abnormalities detected	p	p	p	p	p	p	p	p	p	p	p	p	p	p	p	p	p	p	p	p
Salivary glands																				
No abnormalities detected	p	p	p	p	p	p	p	p	p	p	p	p	p	p	p	p	p	p	p	p

1 = minimal or a couple
2 = mild/slight or a few
3 = moderate or several
4 = marked/severe or many
p = present

four = four such lesions detected
n/a = not applicable
1.5 = between a score of 1 and 2
2.5 = between a score of 2 and 3

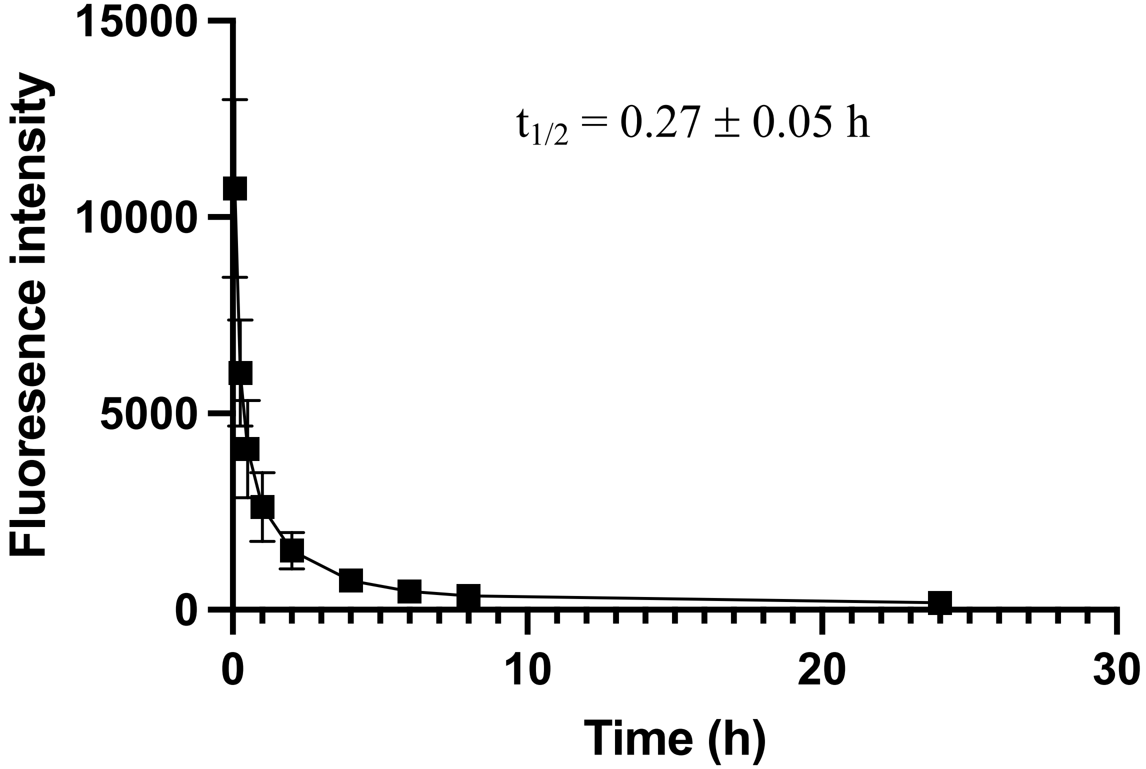

Figure S3. Pharmacokinetic studies of PSMA-1-MMAE-IR700 in male nude mice. Mice received 100 nmol/kg of PSMA-1-MMAE-IR700 through tail vein injection. Blood was taken at different time points. Fluorescence in the blood plasma was then measured (excitation 630 nm, emission at 690 nm). Values are mean±SD of 5 animals.

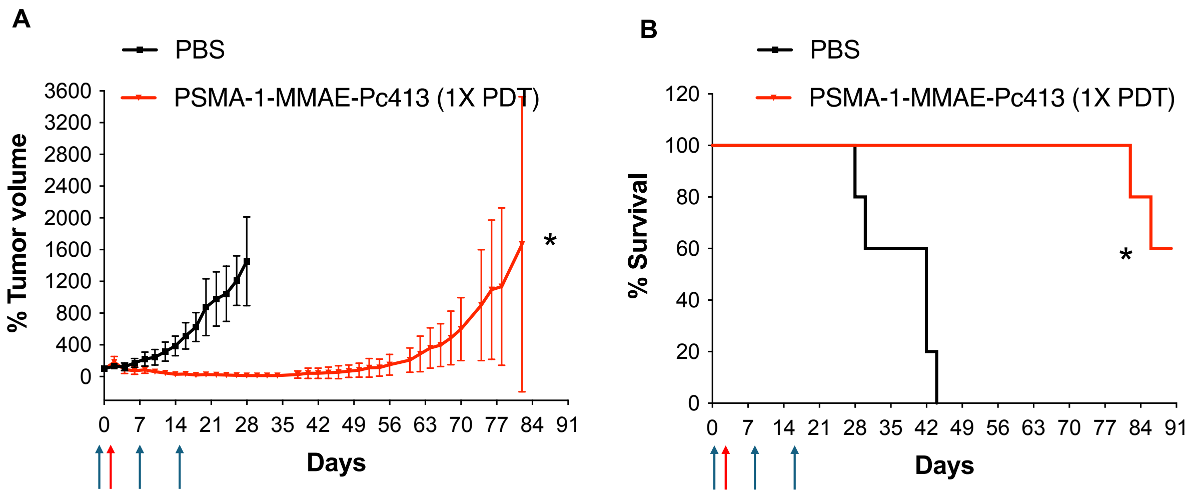

Figure S4. *In vivo* treatment of PC3pip tumors with PSMA-1-MMAE-Pc413 with only a single PDT irradiation (red arrow). Mice received 100 mg/kg of PSMA-1-MMAE-Pc413 every 7 days (blue arrows) with a total of three doses. PDT was performed at 24 h after the first injection only (red arrows), *i.e.* day 1 of the treatment. Blue arrows indicate drug administration time, red arrows indicate PDT application time. (**A**) Tumor growth curves of mice. Values are mean ± SD of 5 animals. *: $p < 0.05$, PSMA-1-MMAE-Pc413 (1X PDT) *vs* PBS. (**B**) Kaplan-Meier survival curves of animals. *: $p < 0.05$, PSMA-1-MMAE-Pc413 (1X PDT) *vs* PBS.

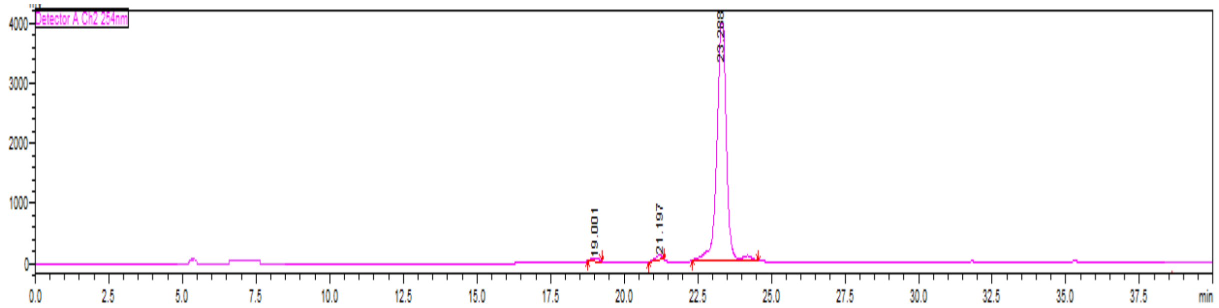

Peak Table | Compound | Group | Calibration Curve

Peak#	Ret. Time	Area	Height	Mark	Conc.	Unit	ID#	Name	Area%
1	19.001	629339	41390	M	0.693				0.693
2	21.197	1447163	93090	M	1.594				1.594
3	23.288	87589102	3934110	M	96.479				96.479
4	24.194	1120226	62237	M	1.234				1.234
Total		90785830	4130826		100.000				100.000

Figure S5. HPLC of PSMA-1-MMAE-Pc413, retention time: 24.19 min, purity: 96.5%. (λ 254 nm, 250 mm x 10 mm Phenomenex Luna C18 column, solvent gradient: 25 mM triethylammonium acetate, pH 7.5 and 70% methanol, reaching 100% of MeOH in 30 min at a flow rate of 2.5 mL/min.)

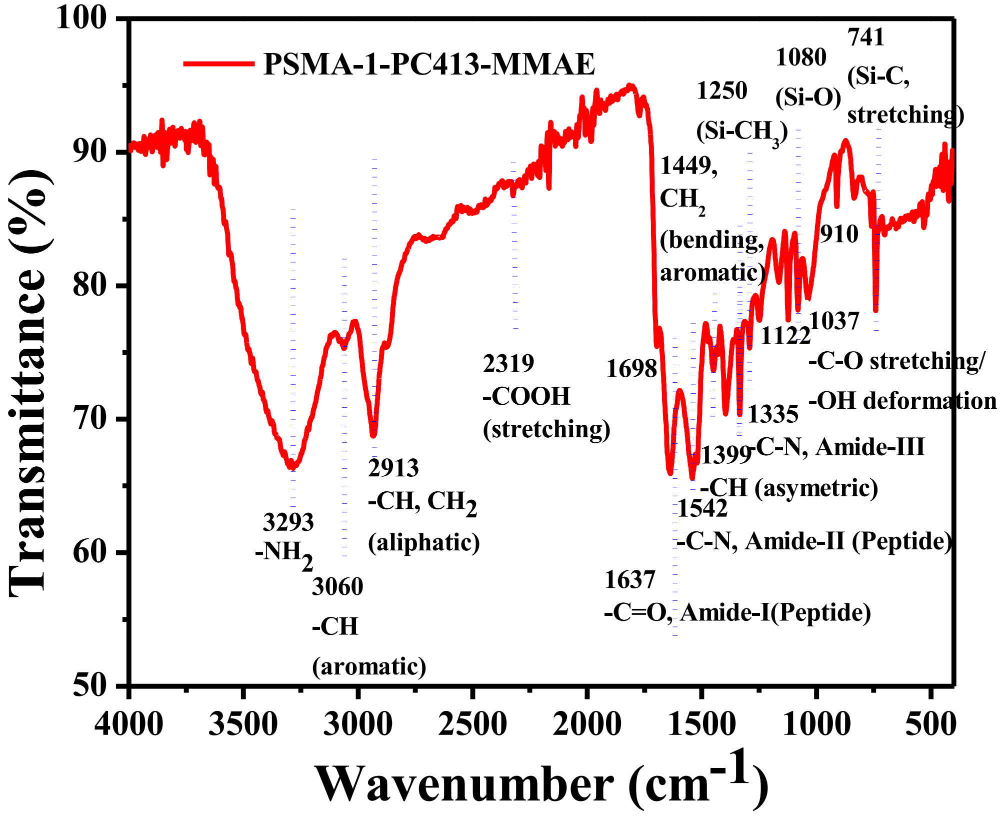

Figure S6. The FT-IR spectrum of PSMA-1-PC413-MMAE. The FT-IR analysis of sample was performed on a JASCO FTIR-4600 spectrometer and used for direct transmission. The bands at 3293, 3060, 2913, and 2319 cm^{-1} has been assigned to $-NH_2$, -CH (aromatic group), -CH, CH_2 (aliphatic stretching group) -COOH, respectively. The bands at 1698, 1637 cm^{-1} corresponded to -$CONH_2$ group of peptides (amide-I). The peaks at 1542, 1449 1399, 1335 cm^{-1} has been assigned to C-N amide II band, $-CH_2$ aromatic bending, -CH asymmetric, C-N amide III groups, respectively. The bands at 1250, 1122, 1080, 910 and 741 cm^{-1} have been assigned to Si-CH_3 bond, Si-O stretching vibration, and Si-C stretching vibration, respectively. This analysis further corroborated the integrity of the structure is retained and successful functionalization of MMAE, PC413 into the peptide group.

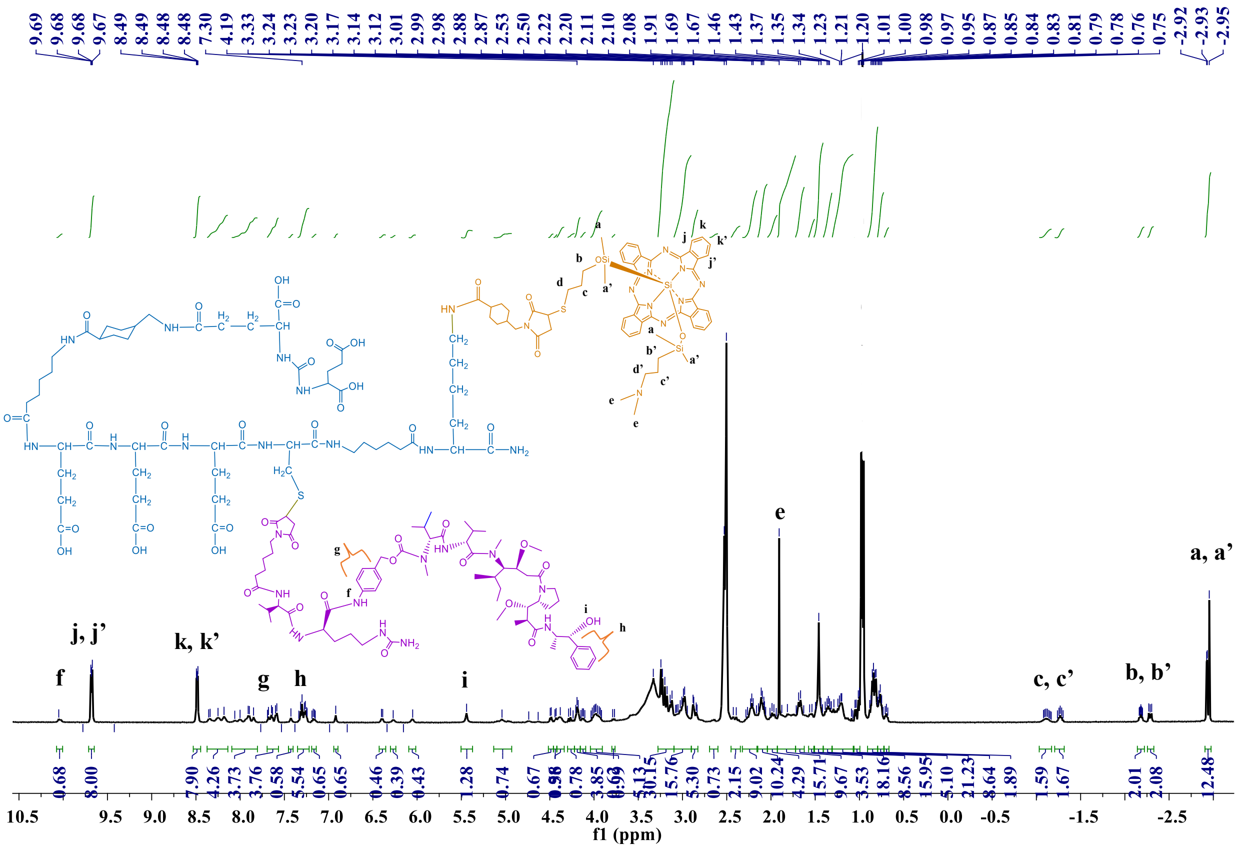

Figure S7. The ^{1}H NMR spectrum of PSMA-1-PC413-MMAE. Conventional ^{1}H spectra were recorded on a 500 MHz Bruker Ascend Avance III HDTM spectrometer in DMSO-*d6*.

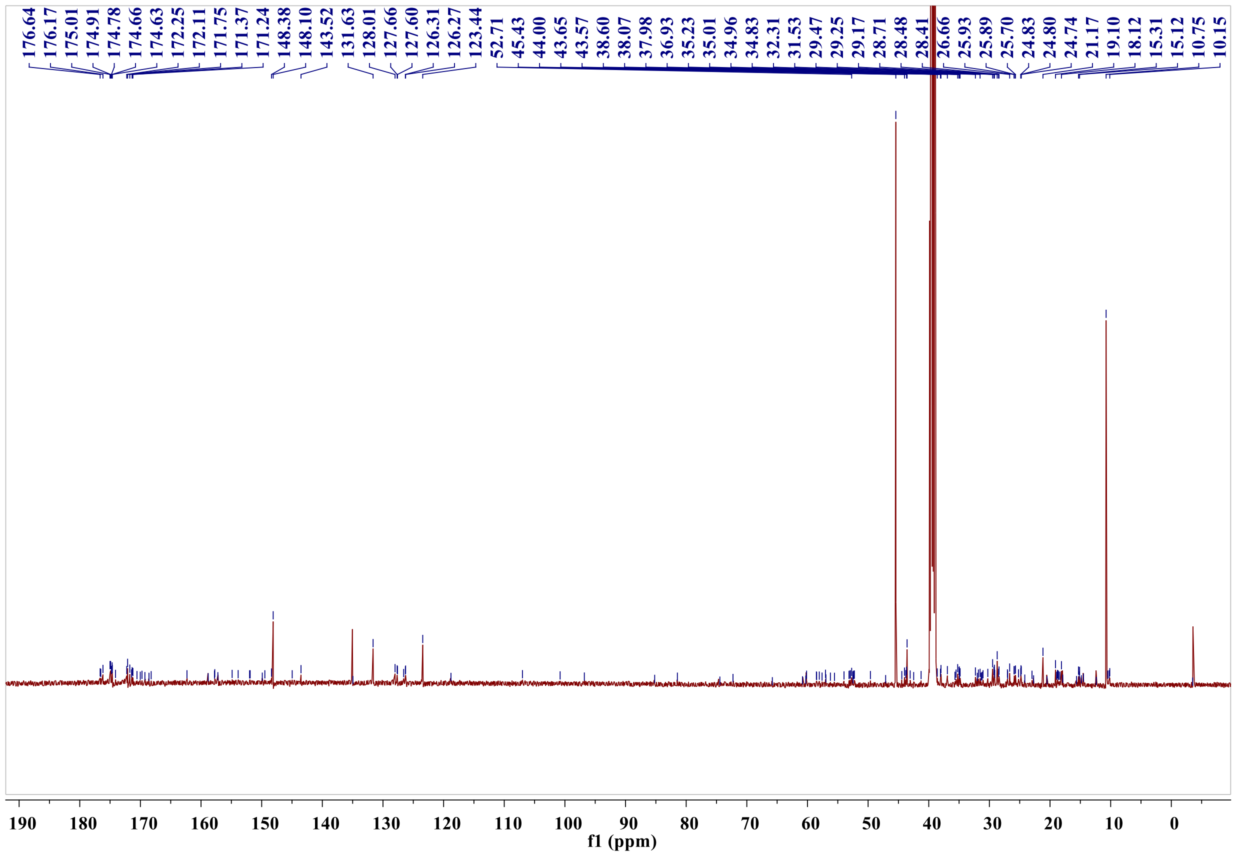

Figure 8. The ^{13}C NMR spectrum of PSMA-1-PC413-MMAE in DMSO-*d6*. ^{13}C NMR spectra were recorded on a 500 MHz Bruker Ascend Avance III HDTM spectrometer (126 MHz for ^{13}C) in DMSO-*d6*.
